# Biosynthesis of dendroketose from different carbon sources using in vitro and in vivo metabolic engineering strategies

**DOI:** 10.1186/s13068-018-1293-7

**Published:** 2018-10-25

**Authors:** Jiangang Yang, Yueming Zhu, Ge Qu, Yan Zeng, Chaoyu Tian, Caixia Dong, Yan Men, Longhai Dai, Zhoutong Sun, Yuanxia Sun, Yanhe Ma

**Affiliations:** 1 National Engineering Laboratory for Industrial Enzymes, Tianjin Institute of Industrial Biotechnology, Chinese Academy of Sciences, Tianjin, 300308 China; 2 School of Pharmacy, Tianjin Medical University, Tianjin, China

**Keywords:** Aldol reactions, Aldolase, Branched-chain sugar, Formaldehyde, Metabolic engineering

## Abstract

**Background:**

Asymmetric aldol-type C–C bond formation with ketones used as electrophilic receptor remains a challenging reaction for aldolases as biocatalysts. To date, only one kind of dihydroxyacetone phosphate (DHAP)-dependent aldolases has been discovered and applied to synthesize branched-chain sugars directly using DHAP and dihydroxyacetone (DHA) as substrate. However, the unstable and high-cost properties of DHAP limit large-scale application. Therefore, biosynthesis of branched-chain sugar from low-cost and abundant carbon sources is essential.

**Results:**

The detailed catalytic property of l-rhamnulose-1-phosphate aldolase (RhaD) and l-fuculose-1-phosphate aldolase (FucA) from *Escherichia coli* in catalyzing the aldol reactions with DHA as electrophilic receptors was characterized. Furthermore, we calculated the Bürgi–Dunitz trajectory using molecular dynamics simulations, thereby revealing the original sources of the catalytic efficiency of RhaD and FucA. A multi-enzyme reaction system composed of formolase, DHA kinase, RhaD, fructose-1-phosphatase, and polyphosphate kinase was constructed to in vitro produce dendroketose, a branched-chain sugar, from one-carbon formaldehyde. The conversion rate reached 86% through employing a one-pot, two-stage reaction process. Moreover, we constructed two artificial pathways in *Corynebacterium glutamicum* to obtain this product in vivo starting from glucose or glycerol. Fermentation with glycerol as feedstock produced 6.4 g/L dendroketose with a yield of 0.45 mol/mol glycerol, representing 90% of the maximum theoretical value. Additionally, the dendroketose production reached 36.3 g/L with a yield of 0.46 mol/mol glucose when glucose served as the sole carbon resource.

**Conclusions:**

The detailed enzyme kinetics data of the two DHAP-dependent aldolases with DHA as electrophilic receptors were presented in this study. In addition, insights into this catalytic property were given via in silico simulations. Moreover, the cost-effective synthesis of dendroketose starting from one-, three-, and six-carbon resources was achieved through in vivo and in vitro metabolic engineering strategies. This rare branched-chain ketohexose may serve as precursor to prepare 4-hydroxymethylfurfural and branched-chain alkanes using chemical method.

**Electronic supplementary material:**

The online version of this article (10.1186/s13068-018-1293-7) contains supplementary material, which is available to authorized users.

## Background

Directed aldol reaction is one of the most powerful carbon–carbon bond-forming procedures in synthetic organic chemistry and enables the concomitant creation of functionalized stereogenic centers and construction of chiral complex polyhydroxylated molecules [1, 2]. Catalytic asymmetric addition of carbon nucleophiles (donor) to ketones (acceptor) is a fundamental approach to construct new tetrasubstituted stereogenic carbon centers. This reaction is synthetically efficient to synthesize chiral tertiary alcohols, which are important building blocks of naturally occurring and artificial biologically active molecules [3]. Aldol addition with ketones as electrophilic receptors is extremely challenging compared with the catalytic enantioselective aldol reaction to aldehydes. Few successful examples have been shown in catalytic aldol reaction to ketones, and they rely on metal catalysts or highly reactive trichlorosilyl enolate of methyl acetate [4–6]. However, all these reactions have a narrow substrate scope and depend on activated ketone acceptors or chiral auxiliaries.

Biocatalyzed aldol additions are attractive because this type of reaction occurs under mild conditions. As such, aldolases are particularly compatible as catalysts in the production of chiral compounds due to high selectivity and catalytic efficiency [7, 8]. A number of aldolases for catalyzing enantioselective aldol additions taking aldehydes as acceptors have been developed [9]. However, to date, only two aldolases that can use ketones as acceptors have been reported. One is pyruvate-dependent aldolase from *Pseudomonas taetrolens*, which exhibits the catalytic ability in aldol addition of pyruvate to a ketone acceptor indole-pyruvic acid and has been used in the stereoselective synthesis of a precursor of monatin [10, 11]. The other one is l-rhamnulose-1-phosphate aldolase (RhaD) from *Bacteroides thetaiotaomicron*. This enzyme catalyzes the aldol reaction between DHAP and several ketones (hydroxyacetone, 1-hydroxybutanone, hydroxypyruvate and l-erythrulose) and gave four branched-chain sugars by coupling an acid phosphatase [12]. The development of several kinds of aldolases that can tolerate ketones as electrophilic receptors is necessary in the asymmetric catalysis field.

Branched-chain sugars, e.g., dendroketose, which belong to a class of rare sugars, are monosaccharides, which rarely exist in nature [13]. Dendroketose was obtained from the polymerization of two molecules of dihydroxyacetone [14, 15]. Typically, dendroketose that contains a tertiary alcohol moiety can be chemically dehydrated to furfural derivatives 4-hydroxymethylfurfural (4-HMF) which showed broad application prospects in preparing fine chemicals [16]. Such conversion is similar to that of fructose to 5-hydroxymethylfurfural (5-HMF) [17]. The synthesis of 2,5-dimethylfuran (DMF) from 5-HMF is a highly attractive route to a renewable fuel [18]. The feasibility of producing 2,4-dimethylfuran (2,4-DMF) or C_9_–C_15_ branched-chain alkanes as liquid transportation fuels from 4-HMF has also been demonstrated [19]. Therefore, developing methods that can synthesize branched-chain sugar is meaningful. The asymmetric aldol addition reactions via DHAP-dependent aldolases are particularly striking strategies in preparation of innovative branched-chain sugars due to the direct and rapid creation of molecular complexity in benign environments [7]. DHAP-dependent aldolases exhibit a strict specificity for the donor DHAP [20]. However, compound DHAP is unstable [21] and currently very expensive. These conditions limit their large-scale application.

In this work, we investigated the catalytic properties of two DHAP-dependent aldolases with ketones as electrophilic receptors. Moreover, we calculated the Bürgi–Dunitz trajectory using molecular dynamics (MD) simulations to reveal the original sources of the catalytic efficiency. Finally, an in vitro multi-enzyme system was designed, and two in vivo artificial pathways were constructed in *Corynebacterium glutamicum* to synthesize dendroketose from one-, three-, and six-carbon resources.

## Results and discussion

### Aldol reactions to DHA catalyzed by DHAP-dependent aldolases

**Fig. 1:**
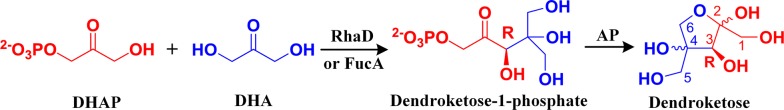
DHAP/DHA-dependent aldolases catalyze the aldol reaction between DHAP and DHA. *RhaD* l-rhamnulose-1-phosphate aldolase, *FucA* l-fuculose-1-phosphate aldolase, *AP* acid phosphatase

DHAP-dependent aldolases have been widely investigated for the synthesis of several new deoxy or phosphorylated sugars and iminocyclitols [22–24]. Naturally, this class of enzyme utilizes DHAP as the donor substrate and accepts a broad range of acceptor aldehydes. Well-known members of this class include FucA, RhaD, fructose 1,6-diphosphate aldolase (FruA), and tagatose 1,6-diphosphate aldolase (TagA). To date, only one kind of DHAP-dependent aldolases has been discovered in catalyzing the aldol reaction between DHAP and ketones and in synthesizing branched-chain sugars [12]. To investigate whether other kinds of DHAP-dependent aldolases showed this catalytic property, RhaD, FucA, TagA and FruA from *Escherichia coli* were used as candidates to catalyze the aldol reaction between DHAP and DHA. Expectedly, both FucA and RhaD enabled the direct aldol addition of DHAP to DHA to form a new compound in combination of acid phosphatase (AP) with conversions of 17% and 73%, respectively (Fig. 1 and Table 1). The RhaD also accepted DHA as nucleophile donor [25]. Therefore, we carried out the aldol reaction with DHA as the sole substrate. An identical product was obtained with a low conversion (7%); however, the reaction needed longer reaction time of 96 h. Naturally, DHAP-dependent aldolases create two new stereogenic centers at C-atoms 3 and 4 [22]. In our work, nuclear magnetic resonance (NMR) analysis showed that both the products obtained from RhaD and FucA have two hydroxymethyl groups at C-4 carbon presenting a branched-chain sugar. RhaD and FucA from *E. coli* have been crystallized and showed a strict 3R-stereoselectivity due to mechanistic requirements [26, 27]. Therefore, those two enzymes shared identical product termed as dendroketose and can be obtained by self-aldolization of DHA using chemical method [19]. Enzymes TagA and FruA from *E. coli* failed to catalyze aldol addition to DHA. Given that RhaD and FucA are class II aldolases, we further measured the catalytic aldol addition to DHA using the class I aldolase, such as fructose 6-phosphate aldolase (FSA) and FruA from rabbit muscle (RAMA). No product was detected when using RAMA and FSA.

**Table 1:** Steady-state kinetic parameters of aldol reactions to DHA catalyzed by RhaD and FucA

	Product	Donor	Acceptor	Conversion (%)	*V*_max_ (U/mg)	*K*_M_ (mM)	*k*_cat_ (s^−1^)	10^3^ × *k*_cat_/*K*_M_ (s^−1^ mM^−1^)
RhaD	l-Fructose	DHAP	l-GAL	92^a^	4.3 ± 0.5	40.8 ± 5.8	20.5 ± 2.5	500 ± 64
	Dendroketose	DHAP	DHA	73	0.86 ± 0.24	23.4 ± 9.69	4.1 ± 0.52	180 ± 40
FucA	l-Tagatose	DHAP	l-GAL	NT	1.5 ± 0.3	54.1 ± 3.6	5.0 ± 0.53	92 ± 13
	Dendroketose	DHAP	DHA	17	0.32 ± 0.13	309.8 ± 45.2	0.45 ± 0.13	1.4 ± 0.5

*NT* not test

^a^This result has been shown in previous paper [38]

### Molecular dynamic simulations provide insights into the catalytic properties of RhaD and FucA

To gain insights into the catalytic properties of RhaD and FucA from *E. coli*, we determined the apparent steady-state kinetic parameters in the reactions of the nucleophile DHAP and acceptor DHA. Substrate l-glyceraldehyde (l-GAL) was evaluated as the control to compare the catalytic properties when using aldehyde and ketone as acceptors. Despite the lower *K*_M_ value of RhaD to DHA (23.4 mM) than that of l-GAL (40.8 mM), the *k*_cat_/*K*_M_ value of RhaD to DHA was threefold lower than that of l-GAL (Table 1). These results indicated that DHA binds to RhaD with higher affinity but with lower catalytic activity than l-GAL. In the case of FucA, the *k*_cat_/*K*_M_ value of FucA to DHA was 65-fold lower than that of FucA to l-GAL. This result indicated that the catalytic activity of FucA toward DHA was significantly lower than that to l-GAL. In terms of *k*_cat_/*K*_M_, clearly, the value of RhaD to DHA was 350-fold higher than that of FucA to DHA. This finding suggested that RhaD is much more efficient in catalyzing the aldol reaction between DHAP and DHA than FucA. According to the *K*_M_ value of FucA in Table 1, the failure of FruA and TagA in catalyzing the aldol addition to DHA was probably due to very low affinity and catalytic efficiency to this substrate.

**Fig. 2:**
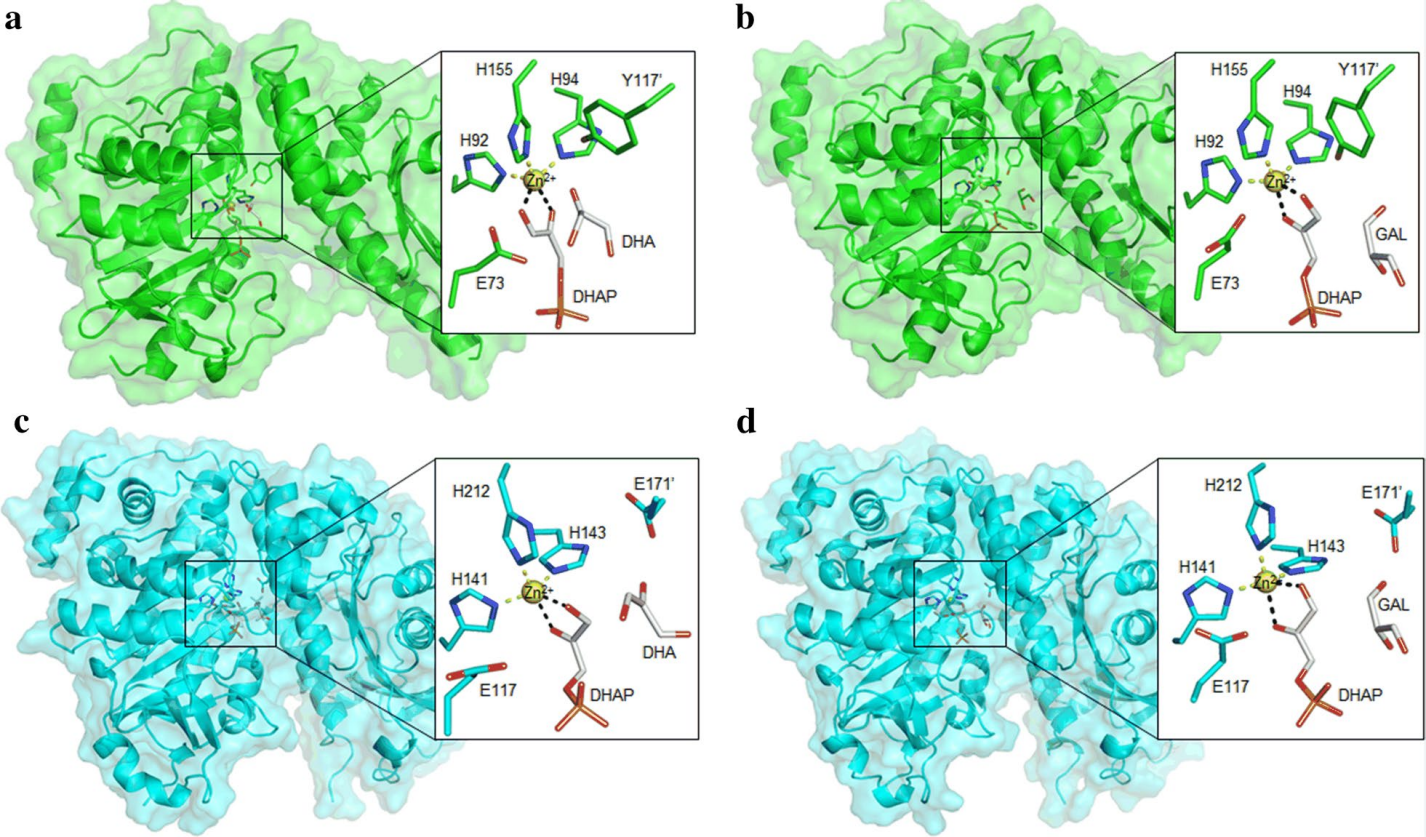
Overview of FucA complex and RhaD complex. **a** FucA–DHAP–DHA; **b** FucA–DHAP–GAL; **c** RhaD–DHAP–DHA; **d** RhaD–DHAP–GAL. Yellow sphere represents zinc ion; white sticks show substrates; green and cyan sticks depict the key residues in FucA and RhaD, respectively

**Fig. 3:**
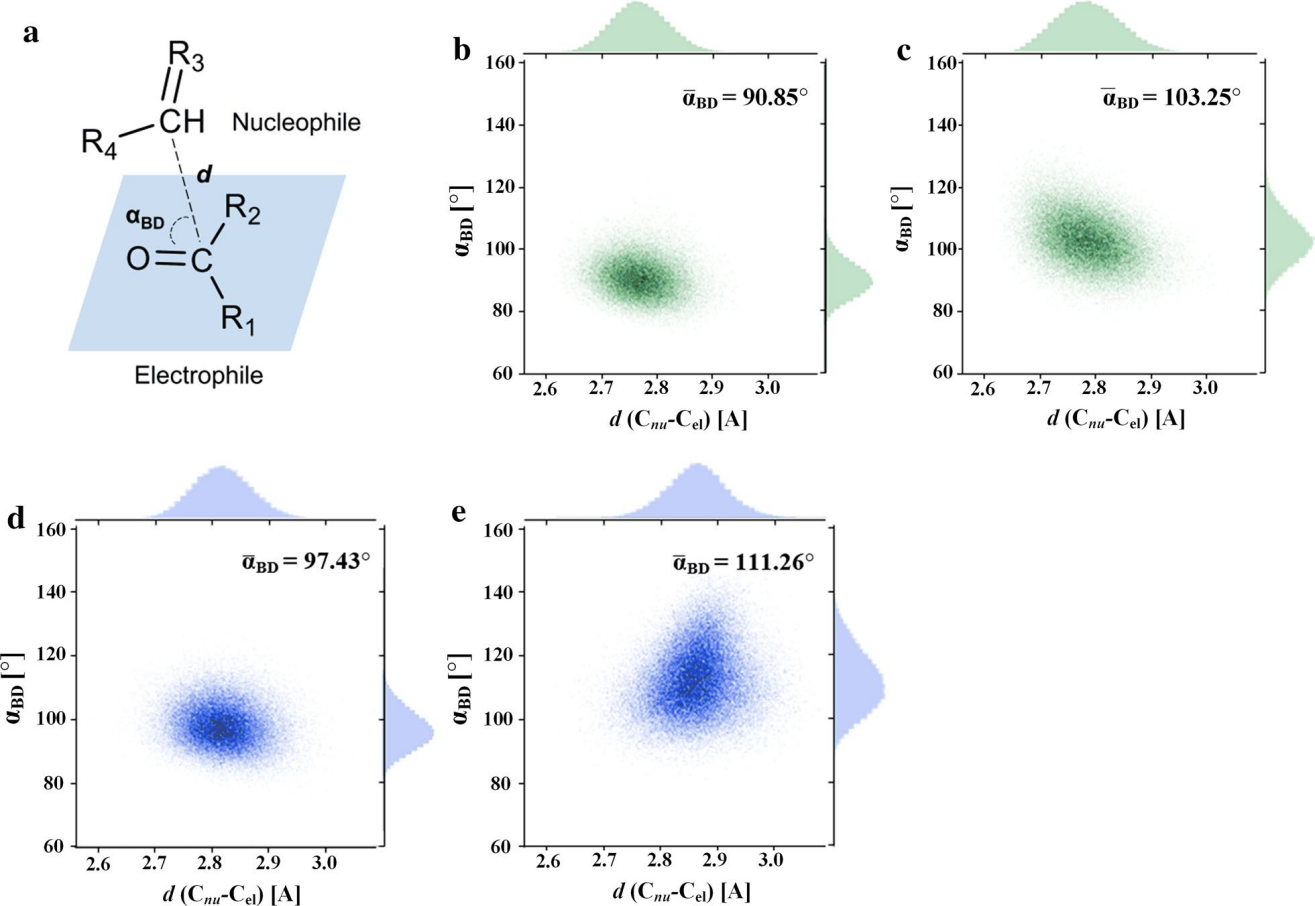
**a** Graphical representation of *α*_BD_. Distance between the carbon of nucleophile and the carbon of electrophile is represented by *d*. Angle (*α*_BD_) versus distance (C_nu_–C_el_) scatter plots for four enzyme–substrate complexes. **b** Complex FucA–DHAP–DHA; **c** complex FucA–DHAP–GAL; **d** complex RhaD–DHAP–DHA; **e** complex RhaD–DHAP–GAL. $\bar{\alpha }_{\text{BD}}$ indicate the average value

Four dimer models of enzyme–substrate complexes (dubbed as “FucA–DHAP–DHA”, “FucA–DHAP–GAL”, “RhaD–DHAP–DHA” and “RhaD–DHAP–GAL”) (Fig. 2) were correspondingly subjected to MD simulations at 300 K (to mimic the experimental conditions) for 100 ns to clarify the possible reasons of the distinct kinetic parameters of FucA and RhaD at the molecular level. Root-mean-square deviation (RMSD) along with root-mean-square fluctuation (RMSF) analysis suggested that the simulated trajectories of all four systems were stable and realizable (Additional file 1: Figures S1 and S2). In organic chemistry theory, the Bürgi–Dunitz angle (*α*_BD_) describes the trajectory of approach of a nucleophile to an electrophile (Fig. 3a). In addition, the value of *α*_BD_ determines whether enzymatic reactions form reactive Michaelis complexes or are arrested. The *α*_BD_ analysis is proved to be a very helpful method in evaluating whether enzymatic reactions are active or not [28, 29]. The ideal *α*_BD_ observed in enzymatic reactions involving the carbonyl groups (e.g., in transaldolase [30], protease [31], and alcohol dehydrogenase [32]) may well differ from the value determined and calculated in organic chemistry (> 105 ± 5°) but will certainly be > 90°. In our study, we analyzed the calculated *α*_BD_ values between the nucleophile DHAP C-atom (C_nu_) and the electrophile *sp*2 carbonyl (C_el_–O) in all four systems (FucA–DHAP–DHA, FucA–DHAP–GAL, RhaD–DHAP–DHA, and RhaD–DHAP–GAL). The catalytic efficiency of FucA and RhaD is dependent on how often the nucleophile and electrophile are present in properly positioned poses and can be reflected at calculated *α*_BD_ value. The average $\bar{\alpha }_{\text{BD}}$ value (90.85°) in FucA–DHAP–DHA system was lower than that of l-GAL as acceptor for FucA ($\bar{\alpha }_{\text{BD}}$ = 103.25°) (Fig. 3b, c). Moreover, the $\bar{\alpha }_{\text{BD}}$ value (97.43°) in RhaD–DHAP–DHA was higher than that (90.85°) of FucA–DHAP–DHA but lower than RhaD–DHAP-l-GAL (Fig. 3d, e). These in silico results were supported by the *k*_*cat*_ value.

### Construction of in vitro multi-enzyme system to produce dendroketose from formaldehyde

**Fig. 4:**
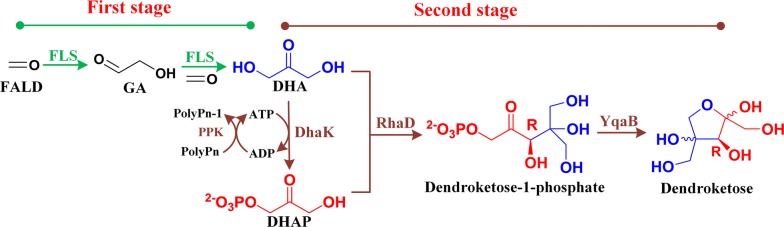
Multi-enzyme reaction systems for dendroketose production from FALD. *PolyPn* polyphosphate, *FLS* formolase, *DhaK* DHA kinase, *RhaD* l-rhamnulose-1-phosphate aldolase, *YqaB* fructose-1-phosphatase, *PPK* polyphosphate kinase

One-carbon compounds as a low-cost, abundant feedstock option have been recently drawing attention in energy and chemical fields [33]. The bioconversion of one-carbon compounds into high-value products is under investigation [34, 35]. Here, we attempted to construct a multi-enzyme system to in vitro synthesize dendroketose with formaldehyde (FALD) as substrate. This system comprised five enzymes, such as formolase (FLS), DHA kinase (DhaK), RhaD, fructose-1-phosphatase (YqaB), and polyphosphate kinase (PPK) (Fig. 4). The enzyme FLS was a computationally designed enzyme with benzaldehyde lyase from *Pseudomonas fluorescens* as starting point. FLS catalyzes the continuous carboligation of FALD to DHA with thiamine pyrophosphate (TPP) as cofactor [36]. DHA phosphorylation catalyzed by DhaK with adenosine triphosphate (ATP) as cofactor contributes in obtaining DHAP. To recycle using ATP, an ATP regeneration system based on PPK and polyphosphate was introduced into the reaction system. Enzyme RhaD catalyzed the aldol addition of the resulting DHAP to DHA to give dendroketose-1-phosphate. The latter was then dephosphorylated by YqaB to synthesize dendroketose (Fig. 4). Along this line, the FLS, DhaK from *Citrobacter freundii* [37], RhaD, YqaB from *E. coli* [38], and PPK from *Rhodobacter sphaeroides* [39] were chosen to construct this multi-enzyme reaction system. Those five enzymes were individually expressed in *E. coli* BL21(DE3). The key information including the Uniprot/Genebank number and enzyme activity for those enzymes is summarized in Table 2. The specific activities of purified enzymes for FLS, RhaD and PPK were 0.09, 0.27 and 18.2 U/mg, respectively.

**Table 2:** The key information of enzymes used in multi-enzyme system

Enzyme	Resource	Uniprot/genebank	Specific activities (U/mg)	References
l-Rhamnulose-1-phosphate aldolase (RhaD)	*Escherichia coli*	P32169	0.27	This study
Fructose-1-phosphatase (YqaB)	*Escherichia coli*	P77475	0.6	[38]
Formolase (FLS)^a^	*Pseudomonas fluorescens*	P51853^a^	0.09	This study
DHA kinase (DhaK)	*Citrobacter freundii*	P45510	22	[37]
Polyphosphate kinase (PPK)	*Rhodobacter sphaeroides*	AWD23073.1	18.2	This study

^a^The enzyme FLS was a mutant of benzaldehyde lyase (BAL) (Uniprot P51853) at A28I, A394G, G419N, and A480W substitution

**Fig. 5:**
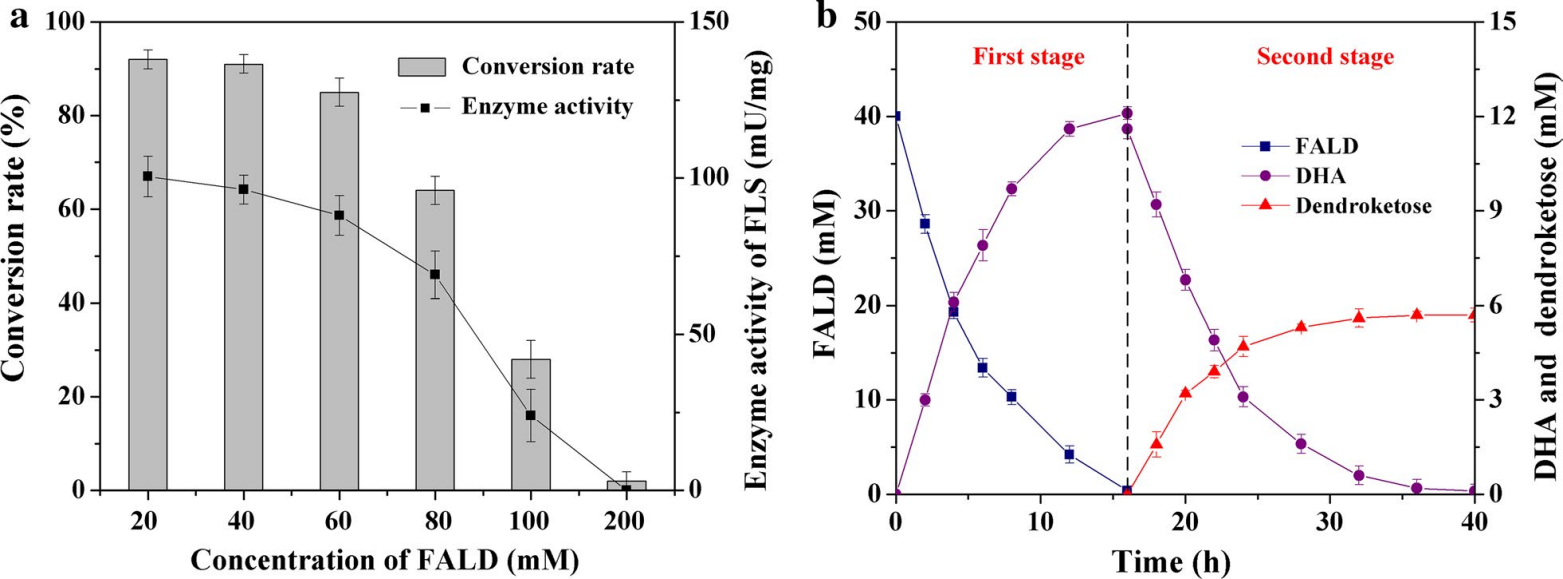
**a** The influence of FALD concentration on FALD conversion and FLS enzyme activity. The conversion rate was calculated with the ratio of DHA formation amount to initial concentration of FLAD. One unit of enzyme activity was defined as the enzyme amount catalyzing the formation of 1 μmol of total DHA and GA per min. **b** Time course of one-pot, two-stage cascade reaction process to synthesize dendroketose from FALD; mean and error bars were calculated based on triplicate experiments

In our multi-enzyme system, the conversion of FALD to DHA was the key step. When FLS catalyzes this carboligation reaction, a small amount of intermediate product glycolaldehyde (GA) was produced [36]. The aldolase RhaD not only converts DHAP and DHA to dendroketose but also catalyzes the aldol addition to substrate FALD and intermediate GA to d-erythrulose and l-xylulose, respectively [40]. We initially mixed the purified enzymes of FLS, DhaK, RhaD, YqaB, and PPK into one-pot reaction medium. However, a mixture of l-xylulose (68%), d-erythrulose (11%) and dendroketose (31%) was obtained (data no shown). In our previous study, one-pot, two-stage reaction process was used to decrease the byproduct formation and increase the product yield during the multi-enzyme cascade reaction with FALD as the sole substrate [34]. Here, this one-pot, two-stage reaction process was again employed. In the first stage, the high conversion of FALD to DHA catalyzed by FLS should be achieved. In the second stage, four other enzymes converted the resulting DHA to dendroketose (Fig. 4). Accordingly, we mixed FLS (1.5 U, 16 mg) and 0.1 mM TPP at different concentration of FALD (20, 40, 60, 80, 100, 200 mM) in the reaction medium and performed the reaction at 30 °C for 16 h in the first stage. The conversion rate of DHA maintained high level (> 90%) at FALD concentration of 20 or 40 mM. However, this rate decreased when FALD concentration was equal to or higher than 60 mM, and no conversion was observed at 200 mM FALD (Fig. 5a). We further measured the enzyme activity of FLS under different FALD concentration. The catalytic ability of FLS indeed decreased when FALD concentration is higher than 60 mM and was absolutely inactivated at 200 mM. Those results indicated that higher FALD concentration inhibited the enzyme activity of FLS and decreased the formation of DHA.

When 40 mM FALD was used, the system produced 12.1 mM DHA with a conversion rate of 91%. This value was calculated with the ratio of DHA formation amount to initial concentration of FLAD. The GA has not been detected in this system. This result was identical to the previous study for which DHA was the primary product at high concentrations of FALD (> 10 mM) [41]. In the second stage, the individually purified enzymes DhaK, RhaD, YqaB, PPK, 10 mM polyphosphate, 0.5 mM ATP and 5 mM MgSO_4_ were added into the reaction system. After reaction for another 24 h, this system produced 5.7 mM (1.03 g/L) dendroketose with a conversion of 86%, which was calculated with the ratio of dendroketose formation amount to initial concentration of FLAD (Fig. 5b). We also detected a small amount of d-erythrulose (~ 0.5 mM) which was derived from the aldol reaction between DHAP and FALD. In this multi-enzyme system, the enzyme activity of FLS to FALD was low for DHA production. Combination of mutational hot spots analytical method and site-saturated mutagenesis strategy has increased the catalytic efficiency of FLS to acetaldehyde for 72.9% [42]. This protein engineering strategy showed the application potential in improving catalytic efficiency of FLS to FALD and further in increasing the production efficiency of dendroketose in the multi-enzyme system.

### Pathway design and strain engineering to produce dendroketose from glycerol

**Fig. 6:**
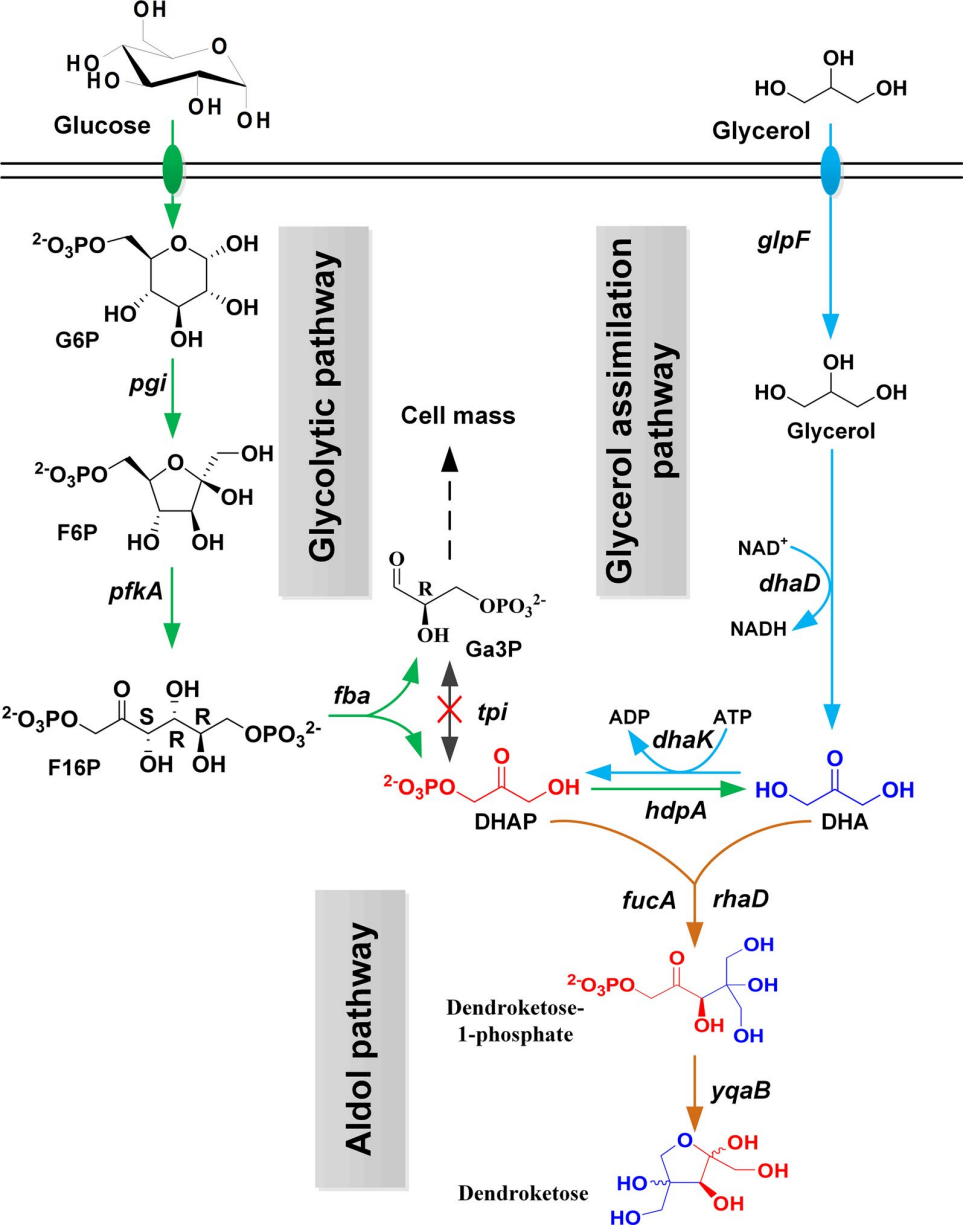
Pathway design and strain engineering in *C. glutamicum* to synthesize dendroketose from glycerol and glucose. *glpF* glycerol facilitator, *dhaD* glycerol dehydrogenase, *dhaK* ATP-dependent dihydroxyacetone kinase, *G6P* glucose 6-phosphate, *F6P* fructose 6-phosphate, *F16P* fructose 1,6-phosphate, *Ga3P* d-glyceraldehyde 3-phosphate, *pgi* encoding glucose-6-phosphate isomerase, *pfkA* encoding 6-phosphofructokinase, *fba* encoding fructose-bisphosphate aldolase, *tpi* encoding triosephosphate isomerase, *hdpA* encoding a HAD superfamily phosphatase, *rhaD* encoding l-rhamnulose-1-phosphate aldolase, *fucA* encoding l-fuculose-1-phosphate aldolase, *yqaB* encoding fructose-1-phosphatase

Glycerol as a major byproduct in biodiesel industry has been considered an abundant and cost-effective feedstock for the production of value-added bioproducts [43–45]. This carbon resource is especially suitable for the production of dendroketose because the conversion of glycerol to DHAP and DHA needs only two enzymatic catalytic reactions which are catalyzed by glycerol dehydrogenase (GDH) and DhaK, respectively (Fig. 6). Here, we investigated the production of dendroketose with glycerol as feedstock through metabolic engineering of *C. glutamicum*, a Gram-positive soil bacterium generally recognized as safe status. In the previous study, the aldol reaction pathway (pXRTY) based on RhaD and YqaB [33] and glycerol assimilation pathway (pEFDK) based on GlpF, glycerol dehydrogenase from *Klebsiella pneumoniae* (DhaD) and DhaK has been combined in wide-type strain, resulting in strain WT(pXRTY/pEFDK) [46]. This strain was initially cultured in CGXII minimal salt medium containing 220 mM glycerol and 110 mM DHA. After fermentation for 48 h, 5.6 g/L (31.1 mM) dendroketose was produced and DHA was absolutely consumed. We still detected 174.5 mM glycerol in the medium. As a result, the dendroketose yield was 0.68 mol/mol glycerol. This value was calculated with the ratio of formation of dendroketose to consumption of glycerol (Table 3). When this train was cultured with 220 mM glycerol as sole feedstock, the production decreased sevenfold. This result was probably due to more carbon flux into biomass but less into desired product.

**Table 3:** Synthesis of dendroketose using different carbon sources

Strains	Medium	Substrates	Production (g/L)	Yield (mol/mol)
WT(pXRTY/pEFDK)^a^	CGXII	Glycerol and DHA	5.6 ± 0.4	0.68 ± 0.03
WT(pXRTY/pEFDK)^b^	CGXII	Glycerol	0.8 ± 0.1	0.05 ± 0.01
SY6(pXRTY/pEFDK)^b^	CGXII	Glycerol	1.2 ± 0.2	0.46 ± 0.02
SY6(pXRTY/pEFDK)^b^	BHI	Glycerol	6.4 ± 0.4	0.45 ± 0.02
WT(pXRTYH)^c^	CGXII	Glucose	ND	–
SY6(pXRTYH)^c^	CGXII	Glucose	36.3 ± 1.3	0.46 ± 0.02
SY6(pXFucTYH)^c^	CGXII	Glucose	8.2 ± 0.6	0.12 ± 0.02

*ND* not detected

^a^The initial concentration of glycerol and DHA was 220 mM and 110 mM, respectively

^b^The initial concentration of glycerol was 220 mM

^c^The initial concentration of glucose was 220 mM. The fermentation was carried out for 48 h. Mean and standard deviation were calculated based on triplicate experiments

**Fig. 7:**
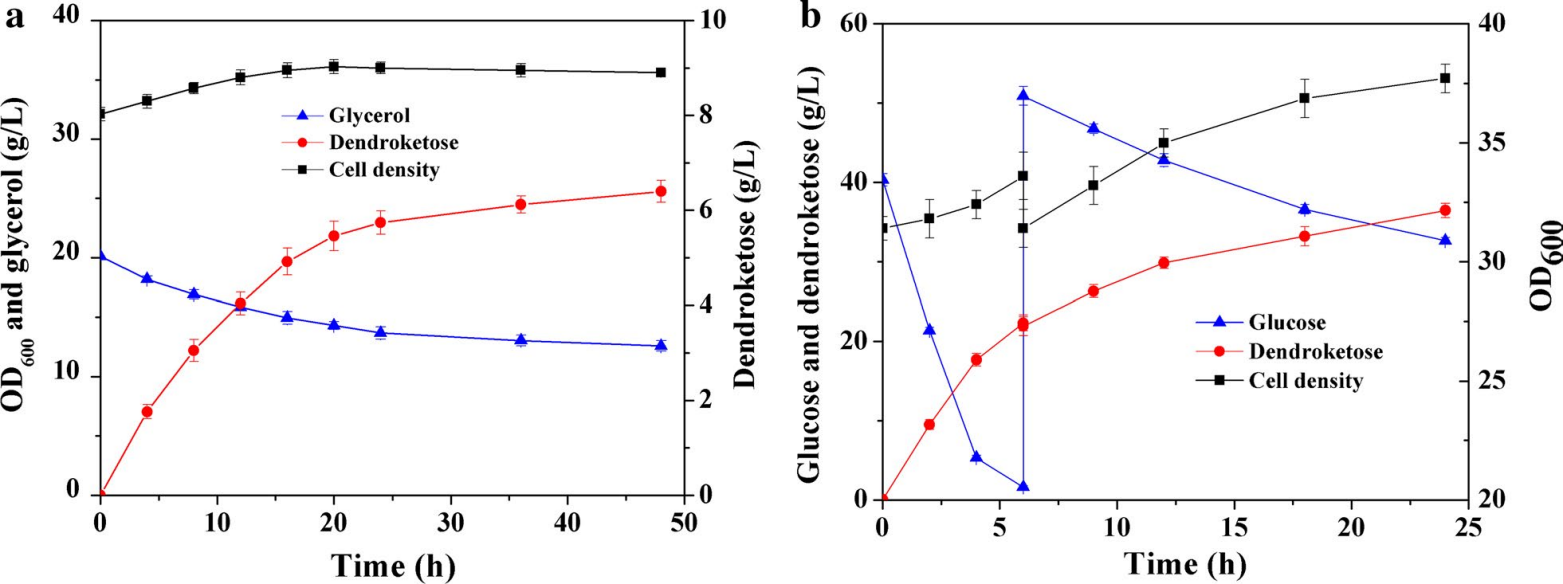
**a** Fermentation of strain SY6(pXRTY/pEFDK) to produce dendroketose from glycerol. The strain was cultivated in BHI-rich medium containing 220 mM glycerol. **b** Fermentation of strain SY6(pXRTYH) to produce dendroketose from glucose. The initial concentration of glucose concentration was 220 mM, and additional 220 mM glucose was supplemented into the reaction medium after 6 h to increase the production. Mean and error bars were calculated based on triplicate experiments

The enzyme triosephosphate isomerase (TPI) catalyzes the isomerization of DHAP to glyceraldehyde 3-phosphate (Ga3P) [47] and then direct carbon flux to biomass. Blockage of this reaction would increase DHAP accumulation and decrease the biomass formation (Fig. 6). Along this line, we constructed strain SY6(pXRTY/pEFDK) by transferring plasmids pXRTY and pEFDK into strain SY6, in which the gene *tpi* was eliminated. When this strain was cultivated in CGXII medium with 220 mM glycerol as the sole carbon source, 1.2 g/L (6.7 mM) dendroketose was produced. This value was 50% higher than that of strain WT(pXRTY/pEFDK). However, the production was still far from satisfactory. Second carbon source permitting the generation of ATP and nicotinamide adenine dinucleotide for cell growth should be co-utilized to increase the efficiency of the glycerol assimilation. Accordingly, strain SY6(pXRTY/pEFDK) was cultured in BHI-rich medium containing the same concentration of glycerol. After fermentation for 48 h, this strain produced 6.4 g/L (35.6 mM) dendroketose and maintained 141 mM glycerol in the medium (Fig. 7a and Table 3). The maximum theoretical yield for dendroketose production with glycerol as substrate is 0.5. In this work, the yield reached 0.45 mol/mol glycerol, representing the 90% of the maximum theoretical value. We also detected small amount of DHA (4.6 mM) in the medium.

### Metabolic engineering of dendroketose production from glucose

Glucose as the most commonly used feedstock can be converted to DHAP via glycolytic pathway in vivo. Enzyme HdpA from *C. glutamicum* belongs to an HAD super family phosphatase and catalyzes the dephosphorylation of DHAP to DHA [48]. In this case, DHAP and DHA would be obtained from the glucose. In this study, we attempt to synthesize dendroketose with glucose as carbon resource through strain engineering of *C. glutamicum* (Fig. 6). Firstly, the TPI should be inactivated to efficiently accumulate DHAP in vivo. Secondly, the gene *hdpA* was overexpressed. Thirdly, the artificial aldol pathway in combination with aldolase RhaD and YqaB from *E. coli* was introduced to convert the accumulated DHAP and DHA to dendroketose. Accordingly, we constructed an engineered *C. glutamicum* strain SY6(pXRTYH), wherein the gene *tpi* was deleted and genes of RhaD, YqaB, and HdpA were overexpressed via plasmid pXRTYH (Table 4). Similarly, strain SY6(pXFucTYH) was constructed via replacement of RhaD with FucA. We have not detected the production of dendroketose in strain WT(pXRTYH) because of scarce accumulation of DHAP in wide-type strain [33]. Fermentation of strain SY6(pXRTYH) gave 36.3 g/L of dendroketose with a yield of 0.46 mol/mol glucose within 24 h (Fig. 7b and Table 3), whereas strain SY6(pXFucTYH) only produced 8.2 g/L of dendroketose (Additional file 1: Figure S4). The production would further increase during fermentation optimizations.

**Table 4:** Strains and plasmids used in this study

Strains and plasmids	Genotype and properties	Source or references
Strain		
*E. coli* DH5α	*supE44 ΔlacU169 (φ80 lacZΔM15) hsdR17 recA1 endA1 gyrA96 thi*-*1 relA1*	Novagen
*E. coli* BL21 (DE3)	*F*-*ompT hsdSB (Rb*-*mB*-*) gal (λ c I 857 ind1 Sam7 nin5 lacUV5 T7gene1) dcm (DE3)*	Novagen
*C. glutamicum*	Wild-type strain	ATCC13032
SY6	Gene *tpi* knock-out in *C. glutamicum* 13032	[40]
WT(pXRTYH)	Wide-type containing plasmid pXRTYH	This study
SY6(pXRTYH)	Strain SY6 containing plasmid pXRTYH	This study
SY6(pXFucTYH)	Strain SY6 containing plasmid pXFucTYH	This study
WT(pXRTY/pEFDK)	Wide-type containing plasmid pXRTY and pEFDK	[46]
SY6(pXRTY/pEFDK)	Strain SY6 containing plasmid pXRTY and pEFDK	This study
Plasmid		
pET21a(+)	Expression vector, *Ap*^R^	Invitrogen
pET21-RhaD	pET21a(+) derivative carrying gene of *rhaD*	[40]
pET21-FucA	pET21a(+) derivative carrying gene of *fucA*	This study
pET21-FLS	pET21a(+) derivative carrying gene of *FLS*	[34]
pET21-YqaB	pET21a(+) derivative carrying gene of *yqaB*	[38]
pET21-DhaK	pET21a(+) derivative carrying gene of *DhaK*	This study
pXRTY	pXMJ19 derivative carrying gene of *rhaD* and *yqaB*	[40]
pEFDK	pECXK99E derivative carrying gene of *glpF*, *dhaD* and *dhaK*	[46]
pXRTYH	pXRTY derivative carrying gene of *hdpA*	This study
pXFucTYH	pXFucTY derivative carrying gene of *hdpA*	This study

## Conclusion

In summary, the detailed catalytic property of RhaD and FucA from *E. coli* in direct aldol addition using DHA as electrophile acceptor was characterized. The Bürgi–Dunitz trajectory calculated by MD simulations has been applied to reveal the catalytic efficiency difference of those two aldolases. Furthermore, we provided a green and environment-friendly biocatalytic approach to synthesize a rare branched-chain sugar dendroketose directly from FALD with high conversion rate. The synthesis of dendroketose from renewable feedstock glucose or glycerol using an engineered strain was also achieved with high titre and yield. Those advanced routes developed in this study presented low-cost way in producing dendroketose. Chemical dehydration of dendroketose will obtain 4-HMF, which serves as a potential platform molecule in preparing certain biofuels and fine chemicals.

## Methods

### Bacterial strains, plasmids and materials

Compounds DHAP, DHA, l-GAL, AP from potato, isopropyl-β-d-thiogalactopyranoside (IPTG), polyphosphate, ATP, l-fructose, l-tagatose, glycerol, glucose, and antibiotics were purchased from Sigma-Aldrich. All restriction enzymes and DNA ligase were purchased from Novagen (Darmstadt, Germany). Ni–NTA affinity chromatography column was purchased from QIAGEN. The yeast extract and tryptone were purchased from OXOID LID, and brain heart infusion (BHI) was purchased from Becton, Dickinson and Company. All bacterial strains and plasmids are listed in Table 4.

### Vectors and strains construction

The genes *fucA* from *E. coli* and *dhaK* from *Citrobacter freundii* were amplified from genome and cloned into pET-21a(+) to obtain pET21-FucA and pET21-DhaK, respectively. The plasmid pET21-PPK containing the gene of *ppk* from *Rhodobacter sphaeroides* was kindly provided by Professor Chun You in our institute. For the construction of plasmid pXRTYH and pXFucTYH, gene *hdpA* was amplified from *C. glutamicum* 13032 genome. The amplified fragments were ligated into plasmid previous constructed plasmids pXRTY [40] and pXFucTY [46] at the *Sma*I and *Sac*I sites to obtain pXRTYH and pXFucTYH, respectively. The constructed plasmids were then electroporated into the recombinant strain SY6, in which the gene *tpi* has been eliminated, to generate strains SY6(pXRTYH) and SY6(pXFucTYH). Plasmid pEFDK containing genes *glpF*, *dhaD* and *dhaK* [46] and pXRTY were co-transformed into SY6 strain to obtain SY6(pXRTY/pEFDK).

### Recombinant proteins expression and purification

*Escherichia coli* BL21(DE3) strains harboring expression plasmids were cultured at 37 °C in 1 L LB medium containing 100 mg/L ampicillin to an optical density OD_600_ of 0.6. 0.5 mM IPTG was added into the culture to induce protein expression and the temperature was adjusted to 16 °C to avoid inclusion body formation. After incubation for an additional 20 h, cells were harvested, washed twice and suspended in 50 mM triethanolamine (TEA) (pH 7.5) buffer. The suspension cells were then lysed by sonication and centrifuged at 14,000×*g* and 4 °C for 10 min. Clear supernatant was collected and loaded onto an Ni^2+^-NTA-agarose column pre-equilibrated with binding buffer (50 mM TEA buffer, 300 mM NaCl, 20 mM imidazole, pH 7.5). The retained proteins were recovered with elution buffer (50 mM TEA buffer, 300 mM NaCl, 300 mM imidazole, pH 7.5). The eluted fraction containing purified protein was dialyzed to eliminate buffer, salt and imidazole. The purified enzymes were freeze dried using a vacuum pump and stored at − 20 °C.

### Enzyme activity assay

The activity of PPK was assayed in a reaction mixture (200 μL) containing PPK (0.05 mg), 25 mM TEA buffer (pH 7.0), 25 mM DHA, 10 mM ADP, 10 mM polyphosphate and 5 mM MgCl_2_·6H_2_O. After the reaction at 30 °C for 30 min, the reaction was stopped by the addition of 10% H_2_SO_4_ (0.5 μL). Product was determined via high-performance liquid chromatography (HPLC). One unit of enzyme activity was defined as the enzyme amount catalyzing the consumption of 1 μmol DHA per min.

The activity of FLS was assayed in a reaction mixture (200 μL) containing FLS (2 mg), 25 mM TEA buffer (pH 7.0), 25 mM FALD, 1 mM MgSO_4_ and 0.1 mM TPP. After the reaction at 30 °C for 45 min, the reaction was stopped by the addition of 10% H_2_SO_4_ (0.5 μL). One unit of enzyme activity was defined as the enzyme amount catalyzing the formation of 1 μmol of total DHA and GA per min.

### Steady-state kinetic parameters of RhaD and FucA to DHA and l-GAL

Reaction: aldol addition of DHAP to l-GAL. To a solution containing freshly neutralized DHAP (60 mM) and RhaD (0.05 mg powder) in 50 mM TEA buffer pH 7.5 at 25 °C, different amounts of l-GAL (0.2, 0.5, 2, 5, 10, 20, 40, 60 mM) were added. The final volume was 400 μL. Samples (40 μL) were withdrawn at different times (0, 2, 5, 10, 20, 30 min) and the reaction was stopped by the addition of 10% H_2_SO_4_ (0.5 μL). Samples were then analyzed by HPLC to measure the l-GAL consumption. One mmol of l-GAL consumed was equivalent to 1 mmol of l-fructose-1-phosphate formed.

Reaction: aldol addition of DHAP to DHA. To a solution containing freshly neutralized DHAP (60 mM) and RhaD (0.05 mg powder) in 50 mM TEA buffer pH 7.5 at 25 °C, different amounts of DHA (2, 5, 10, 20, 40, 60, 80 mM) were added. The final volume was 400 μL. Samples (40 μL) were withdrawn at different times (0, 2, 5, 10, 20, 30 min) and the reaction was stopped by the addition of 10% H_2_SO_4_ (0.5 μL). Samples were then analyzed by HPLC to measure the DHA consumption. One mmol of DHA consumed was equivalent to 1 mmol of adduct formed.

Reaction: aldol addition of DHAP to l-GAL. To a solution containing freshly neutralized DHAP (60 mM) and FucA (0.1 mg powder) in 50 mM TEA buffer pH 7.5 at 25 °C, different amounts of l-GAL (0.5, 2, 5, 10, 20, 40, 60 mM) were added. The final volume was 400 μL. Samples (40 μL) were withdrawn at different times (0, 2, 5, 10, 20, 30 min) and the reaction was stopped by the addition of 10% H_2_SO_4_ (0.5 μL). Samples were then analyzed by HPLC to measure the l-GAL consumption. One millimole of l-GAL consumed was equivalent to 1 mmol of l-tagatose-1-phosphate formed.

Reaction: aldol addition of DHAP to DHA. To a solution containing freshly neutralized DHAP (60 mM) and FucA (0.1 mg powder) in 50 mM TEA buffer pH 7.5 at 25 °C, different amounts of DHA (2, 5, 10, 20, 40, 60, 80, 100, 200 mM) were added. The final volume was 400 μL. Samples (40 μL) were withdrawn at different times (10, 20, 40, 60, 90, 120, 180 min) and the reaction was stopped by the addition of 10% H_2_SO_4_ (0.5 μL). Samples were then analyzed by HPLC to measure the DHA consumption. One mmol of DHA consumed was equivalent to 1 mmol of adduct formed.

### Aldol reactions with DHAP and DHA as substrates

The reaction mixture (1 mL) contained freshly neutralized 50 mM DHAP solution, 50 mM DHA, 50 mM TEA buffer (pH 7.5) and RhaD (1 mg) or FucA (2 mg). The reaction mixture was transferred to a 1.5-mL Eppendorf tube and shaken at 25 °C and 120 rpm for 24 h. Then, the pH of the mixture was adjusted to 4.5–5.5 using 10% H_2_SO_4_, and 2 U AP was supplemented. The dephosphorylation reaction was performed at 30 °C for another 24 h.

### Molecular modeling

Models of the dimer structures of FucA complex and RhaD complex were generated as follows: the monomer structure of FucA is derived from the previous study (PDB code 4FUA). However, there is no available polymer structure of FucA by searching the PDB database. TM-align program [49] was used to search for FucA homologies; in the top 10 hits ranked by TM-score, 2OPI crystalized in polymers was used as template. In the case of RhaD, the X-ray structure (PDB code 1GT7) was directly used as the basis for dimer creation of RhaD. The coordinates of the donor DHAP and the acceptor DHA/L-GAL in constructed dimers were superimposed with those from the PDB codes 1OJR and 4FUA.

Based on the catalytic mechanism of class II Aldolase, a specific residue in the adjacent monomer (Tyr113′ in FucA, and Glu171′ in RhaD) plays a key role on the protonation of the carbonyl oxygen of ketone acceptors. Therefore, dimer models of enzyme–substrate complexes (dubbed as “FucA–DHAP–DHA”, “FucA–DHAP–GAL”, “RhaD–DHAP–DHA” and “RhaD–DHAP–GAL”) were built to reflect the catalytic mechanism.

During the simulations, a constant force of 10 kcal/mol between the nucleophile C-atom and the electrophile C-atom was constructed via the consideration of Van der Waals’ force. To estimate the stability of 100 ns trajectories of the four systems, root-mean-square deviation (RMSD) for all C_α_ atoms was analyzed and no significant structure difference was observed. Furthermore, root-mean-square fluctuation (RMSF) which could reflect the stability of individual residue of protein was also evaluated; most residues are stable except for the terminations between two monomers.

### Molecular dynamics (MD) simulations

The initial structures used for MD simulation were obtained from modeling analysis. Each apo-protein was protonated at pH 7.5 using H++ webserver. The Amber ff14SB force field was employed for the protein in all the MD simulations [50]. Na^+^ ions were added to neutralize the system, and the TIP3P water model was used to solvate each system, ensuring a solvent layer of at least 10 Å from any point on the protein surface. Charges and parameters for ligands were generated with the Antechamber module using the AM1-BCC charge model along with the amber GAFF force field. The force field of zinc ion and its neighboring atoms (cutoff was set as 2.8 Å) were parameterized using ‘MCPB.py’ modeling, using a hybrid bonded/restrained nonbonded model. As a result, the three histidine residues (H92, H94 and H155 in FucA, and H141, H143 and H212 in RhaD) were attached to zinc ion by coordinate bonds, whereas the two oxygen atoms of donor were attached to zinc ion by applying harmonic restraint (100 kcal/mol). After proper parameterizations and setup, the resulting system’s geometries were minimized (5000 steps for steepest conjugate and 5000 steps for conjugate gradient) to remove poor contacts and relax the system. The systems were then annealed from 0 to 300 K (≈ 27 °C) to mimic experimental temperature under the constant amount of substance (*N*), volume (*V*) and temperature (*T*) (NVT ensemble) for 50 ps. Subsequently, the systems were maintained for 25 ps of density equilibration under constant amount of substance (*N*), pressure (*P*) and temperature (*T*) (NPT ensemble) at constant temperature of 300 K and pressure of 1.0 atm using Langevin-thermostat (ntt = 3) with collision frequency of 2 ps^−1^ and pressure relaxation time of 1 ps. The heating and density equilibrations were carried out with a weak restraint of 20 kcal mol^−1^ Å^−2^ performed on all the residues. The systems were further equilibrated for 250 ps to get well settled pressure and temperature for conformational and chemical analyses. After proper minimizations and equilibrations, a productive MD run of 100 ns was performed for each system. During all MD simulations, the covalent bonds containing hydrogen were constrained using SHAKE algorithm [51], with a MD time step of 2 fs. The trajectory file was written every 1000 steps. All the above MD simulations were performed with GPU version of Amber 16 package. The generated trajectories (interval = 200, a total of 500 frames for each case) were used for the relative binding energy evaluation.

### In vitro cascade reaction

To synthesize dendroketose in vitro, the reaction mixture (2 mL) containing 50 mM TEA (pH 7.5), 40 mM FALD, 0.1 mM TPP, FLS (1.5 U, 16 mg) was initially carried out at 25 °C and 120 rpm for 16 h. Then, DhaK (0.5 U, 0.025 mg), PPK (0.5 U, 0.03 mg), RhaD (0.5 U, 2 mg), YqaB (0.9 U, 1.5 mg), polyphosphate (20 mM), ATP (0.5 mM), and MgSO_4_ (10 mM) were added into the reaction system, and it performed for another 24 h. Samples (100 μL) were captured very two hours, treated with 10% H_2_SO_4_, centrifuged (22,000 rpm, 20 min) and analyzed by HPLC.

### Shake flask scale cultivation

For precultivation of recombinant strain, a single clone was grown in 5 mL of BHI medium. After incubation for approximately 15 h, cells were inoculated into a 500-mL shake flask containing 100 mL BHI medium and cultivated at 25 °C in a rotatory shaker at 220 rpm. When the cell OD_600_ reached 0.8, 1 mM IPTG was added to induce enzyme expression. Subsequently, the cells were harvested by centrifugation (8000×*g*, 10 min, 4 °C) and were suspended in CGXII medium [52]. Then, 50 mL cells were transferred into a 250-mL shake flask with an initial OD_600_ of approximately 30. When appropriate, 10 mg/L chloramphenicol and 25 mg/L kanamycin were added. The fermentation process was carried out at 30 °C and 200 rmp.

If glycerol and DHA were used as substrates, the concentration of glycerol and DHA was assigned to 220 mM and 110 mM, respectively. To produce dendroketose from glucose, 220 mM glucose was supplemented into the medium. For the fed-batch fermentation, 220 mM glucose was supplemented again into the medium after fermentation for 6 h. Samples were collected every 2 h and centrifuged at 14,000×*g* for 20 min. The resulting supernatants were analyzed by HPLC. The desired product was separated by a chromatographic column filled with Ca^2+^ ion exchange resin, identified by a refractive index detector and then collected by a fraction collector. The purified products were analyzed by NMR.

### Analytical methods

Cell density was determined by measuring the optical density at 600 nm (OD_600_) with a UV–Vis spectrophotometer (TU-1901, Persee, Beijing, China). Cell dry weight (CDW, g/L) of *E. coli* was calculated from OD_600_ values using the experimentally determined correlation factor of 0.25 g cells (dry weight [DW])/liter for an OD_600_ of 1. Protein concentrations were determined by the Bradford method using bovine serum albumin as a standard. HPLC system (Agilent 1100 series, Hewlett-Packard) equipped with a refractive index detector and fitted with chromatographic column (Bio-Rad Aminex HPX-87H column or Waters Sugar-Pak I column) was used to qualitative and quantitative analysis of substrates and products.

## Additional file

**Additional file 1:** Figure S1. Root mean square deviations (RMSD) measured during 100 ns MD simulation. **Figure S2.** Root mean squared fluctuations (RMSF) measured during 100 ns MD simulation. The terminations between two monomers are highlighted with yellow box. The monomer size of FucA is 206 aa, whilst 274 aa for RhaD monomer. **Figure S3.** The standard curve of verified dendroketose. **Figure S4.** Fermentation of strains SY6(pXFucTYH) to produce dendroketose from low cost glucose. The initial glucose concentration was 220 mM, and glucose was additional supplemented into the reaction medium at the reaction time for 6 h to increase dendroketose production. **Figure S5.** MS data of dendroketose synthesized by RhaD and AP. **Figure S6.** MS data of dendroketose synthesized by FucA and AP. **Figure S7.** Observed NMR spectra of dendroketose obtained by RhaD. **Figure S8.** Observed NMR spectra of dendroketose obtained by FucA.
