# Supplementary material for: Biosynthesis of dendroketose from different carbon sources using in vitro and in vivo metabolic engineering strategies
Source: Biotechnol Biofuels. 2018 Oct 25;11:290. doi: 10.1186/s13068-018-1293-7 (PMC6202814; doi:10.1186/s13068-018-1293-7)
Supplement: Supplementary file 1 — Additional file 1: Figure S1. Root mean square deviations (RMSD) measured during 100 ns MD simulation. Figure S2. Root mean squared fluctuations (RMSF) measured during 100 ns MD simulation. The terminations between two monomers are highlighted with yellow box. The monomer size of FucA is 206 aa, whilst 274 aa for RhaD monomer. Figure S3. The standard curve of verified dendroketose. Figure S4. Fermentation of strains SY6(pXFucTYH) to produce dendroketose from low cost glucose. The initial glucose concentration was 220 mM, and glucose was additional supplemented into the reaction medium at the reaction time for 6 h to increase dendroketose production. Figure S5. MS data of dendroketose synthesized by RhaD and AP. Figure S6. MS data of dendroketose synthesized by FucA and AP. Figure S7. Observed NMR spectra of dendroketose obtained by RhaD. Figure S8. Observed NMR spectra of dendroketose obtained by FucA. [file 13068_2018_1293_MOESM1_ESM.docx]

**Biosynthesis of** **dendroketose from different carbon sources using *in vitro* and *in vivo* metabolic engineering strategies**

Jiangang Yang^§†^  Yueming Zhu^§†^ Ge Qu^†^ Yan Zeng^†^ Chaoyu Tian^†^ Caixia Dong^‡^ Yan Men^†^ Longhai Dai^†^ Zhoutong Sun*^†^ Yuanxia Sun*^†^ Yanhe Ma^†^

^†^ National Engineering Laboratory for Industrial Enzymes, Tianjin Institute of Industrial Biotechnology, Chinese Academy of Sciences, Tianjin 300308, China.

‡ School of Pharmacy, Tianjin Medical University, Tianjin, China.

E-mail address:

Jiangang Yang: [yang_jg1@tib.cas.cn](mailto:yang_jg1@tib.cas.cn)

Yueming Zhu: [zhu_ym@tib.cas.cn](mailto:zhu_ym@tib.cas.cn)

Ge Qu: [qug@tib.cas.cn](mailto:qug@tib.cas.cn)

Yan Zeng: [zeng_y@tib.cas.cn](mailto:zeng_y@tib.cas.cn)

Chaoyu Tian: [tiancy@tib.cas.cn](mailto:tiancy@tib.cas.cn)

Caixia Dong: [dongcaixia@tmu.edu.cn](mailto:dongcaixia@tmu.edu.cn)

Yan Men: [men_y@tib.cas.cn](mailto:men_y@tib.cas.cn)

Longhai Dai: [dai_lh@tib.cas.cn](mailto:dai_lh@tib.cas.cn)

Zhoutong Sun: [sunzht@tib.cas.cn](mailto:sunzht@tib.cas.cn)

Yuanxia Sun: [sun_yx@tib.cas.cn](mailto:sun_yx@tib.cas.cn)

Yanhe Ma: [ma_yh@tib.cas.cn](mailto:ma_yh@tib.cas.cn)

**Figure S1.** Root mean square deviations (RMSD) measured during 100 ns MD simulation.


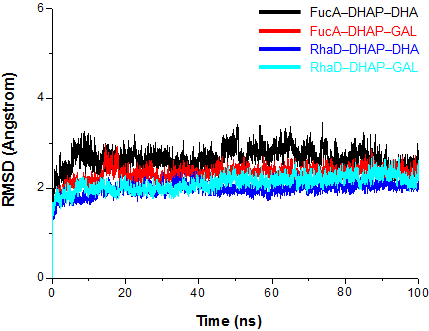


**Figure S2.** Root mean squared fluctuations (RMSF) measured during 100 ns MD simulation. The terminations between two monomers are highlighted with yellow box. The monomer size of FucA is 206 aa, whilst 274 aa for RhaD monomer.


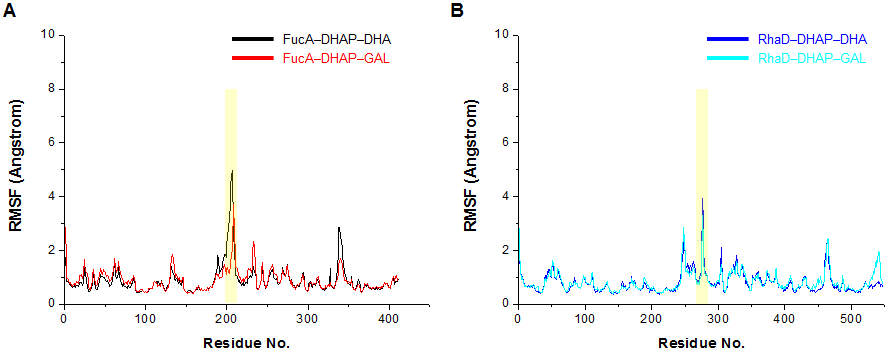


**Figure S3** The standard curve of verified dendroketose.


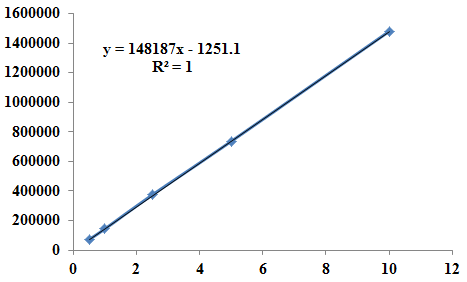


**Figure S4.** Fermentation of strains SY6(pXFucTYH) to produce dendroketose from low cost glucose. The initial glucose concentration was 220 mM, and glucose was additional supplemented into the reaction medium at the reaction time for 6 h to increase dendroketose production.





**MS analysis data**

**Figure S5.** MS data of dendroketose synthesized by RhaD and AP

[M+Na]^+^

Measured m/z value of [**M**+Na]^+^ of dendroketose: 203.0549 was identical with the theoretical value of 203.147.

**Figure S6**. MS data of dendroketose synthesized by FucA and AP

[M+Na]^+^

Measured m/z value of [**M**+Na]^+^ of dendroketose: 203.0550 was identical with the theoretical value of 203.147.

**NMR analysis data**

NMR data of dendroketose was identical to those described in the literature (Deng J, Pan T, Xu Q, Chen MY, Zhang Y, Guo QX, Fu Y. Linked strategy for the production of fuels via formose reaction. Sci Rep. 2014; 3: 1244.).

^1^H- and ^13^C correlations

| No. | d_C_ (ppm) | d_H_ (ppm) |
| --- | --- | --- |
| 1 | 62.76 | 3.535 (H1a, 1H, s) 3.530 (H1b, 1H, s) |
| 2 | 103.18 |  |
| 3 | 70.58 | 3.95 (H3, 1H, s) |
| 4 | 78.24 |  |
| 5 | 73.00 | 3.91 (H5, 2H, s) |
| 6 | 63.93 | 3.57(H6a/H6b, 2H, s) |

Two- or three-bond ^1^H- and ^13^C correlations

| Proton | Proton correlation |
| --- | --- |
| H1a | C-2 |
| H1b | C-2 |
| H3 | C-1 |
| H5 | C-6, C-3, C-4, C-2 |
| H6a/H6b | C-3, C-4, C-5 |

## Figure S7. Observed NMR spectra of dendroketose obtained by RhaD.

**a)** ^1^H;  **b)** ^13^C; **c)** 2D HMBC;  **d)** 2D HSQC.


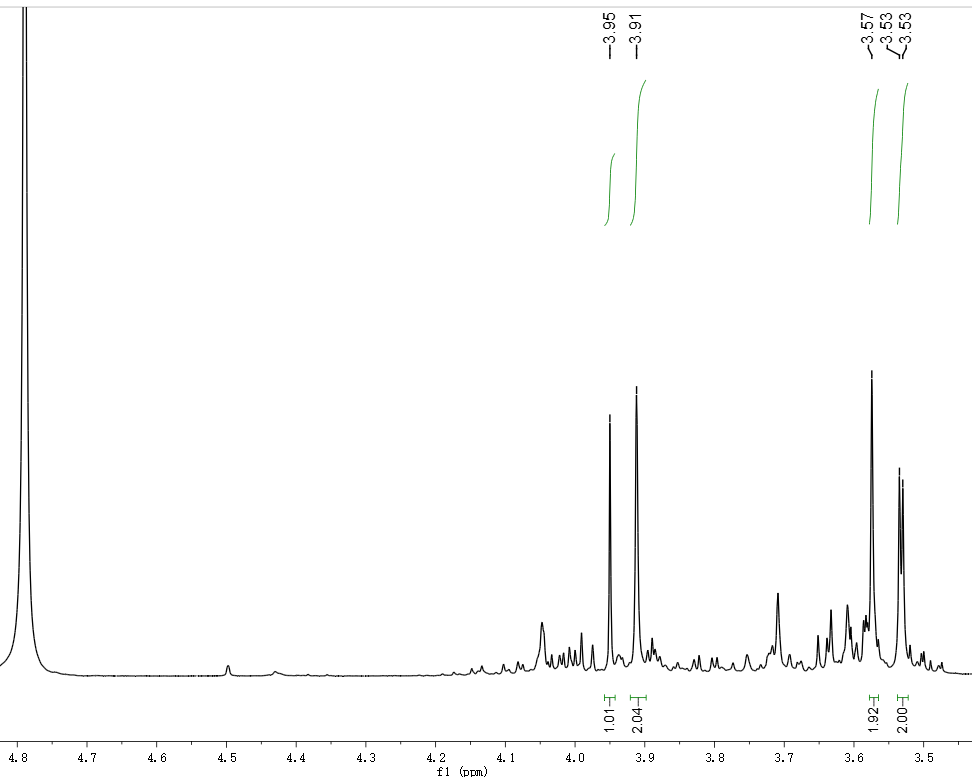


**a）**


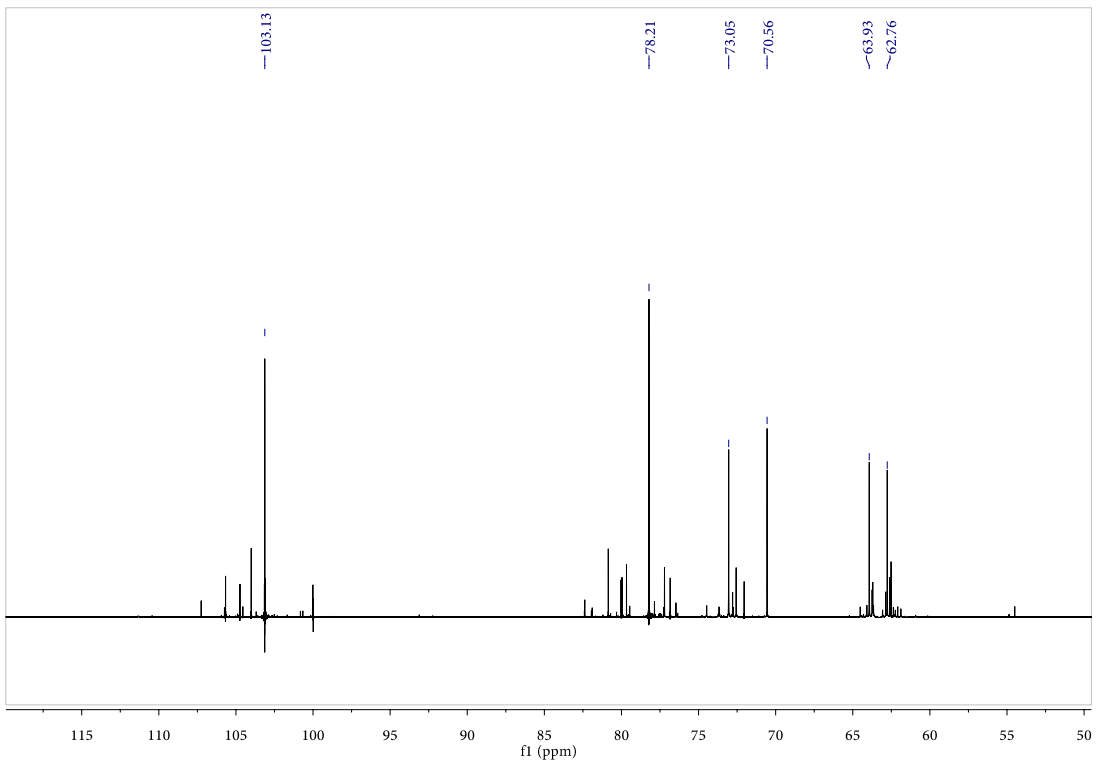


**b)**


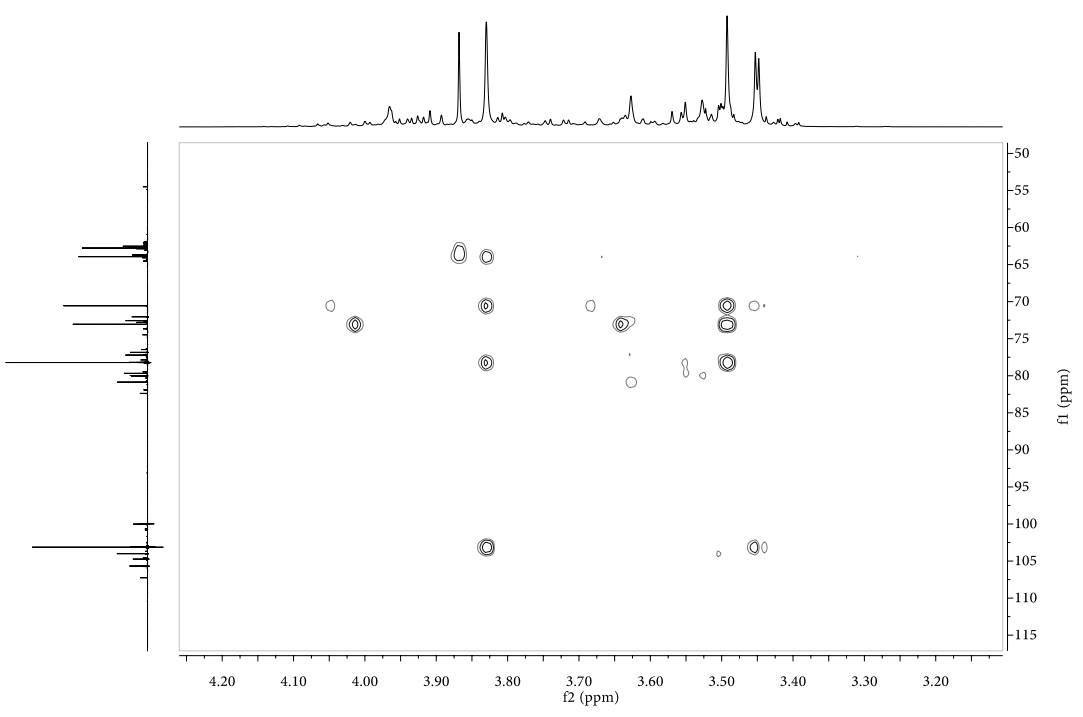


**c)**


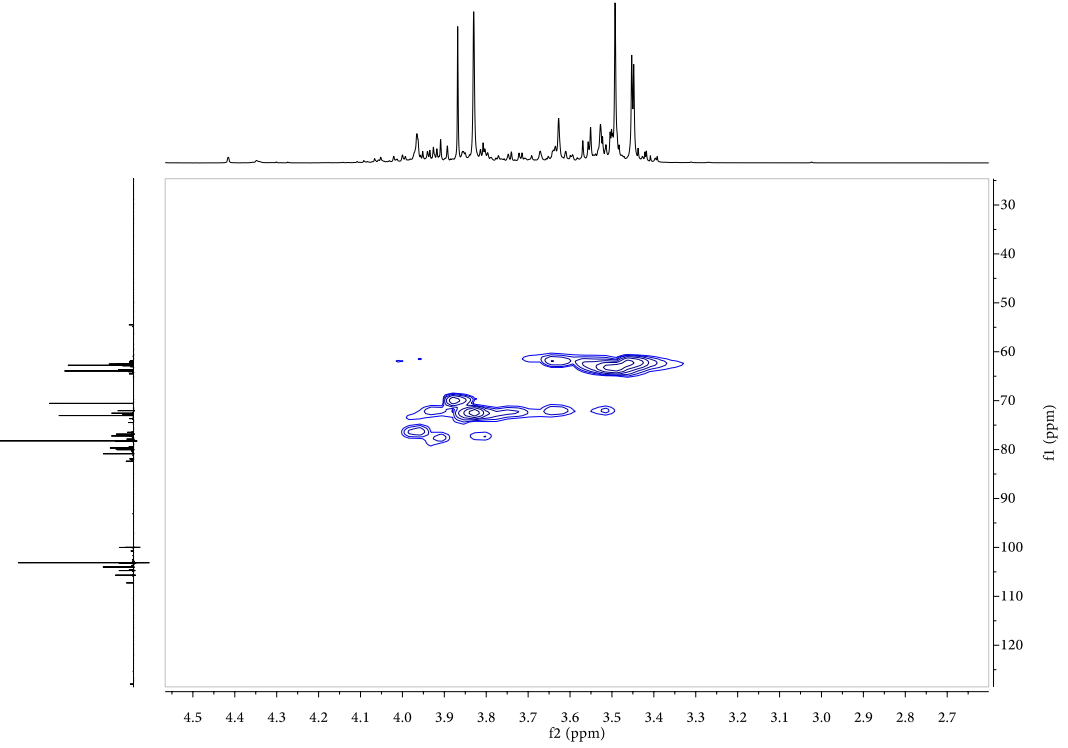


**d)**

## Figure S8. Observed NMR spectra of dendroketose obtained by FucA.

**a)** ^1^H;  **b)** ^13^C; **c)** 2D HMBC;  **d)** 2D HSQC.


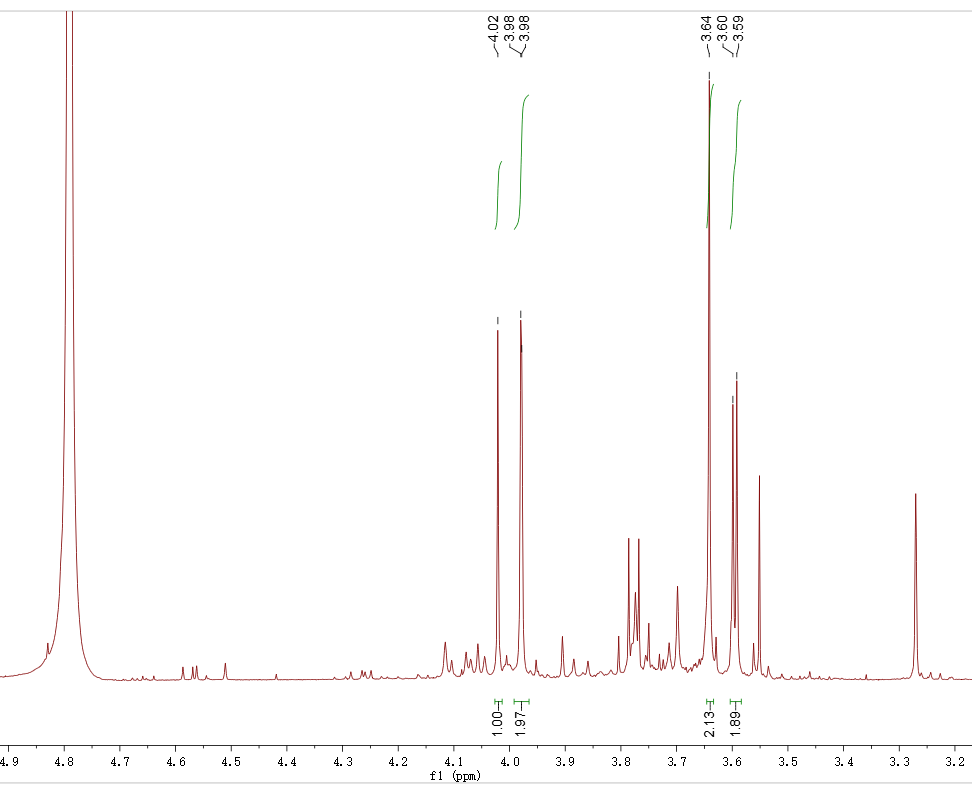


**a)**


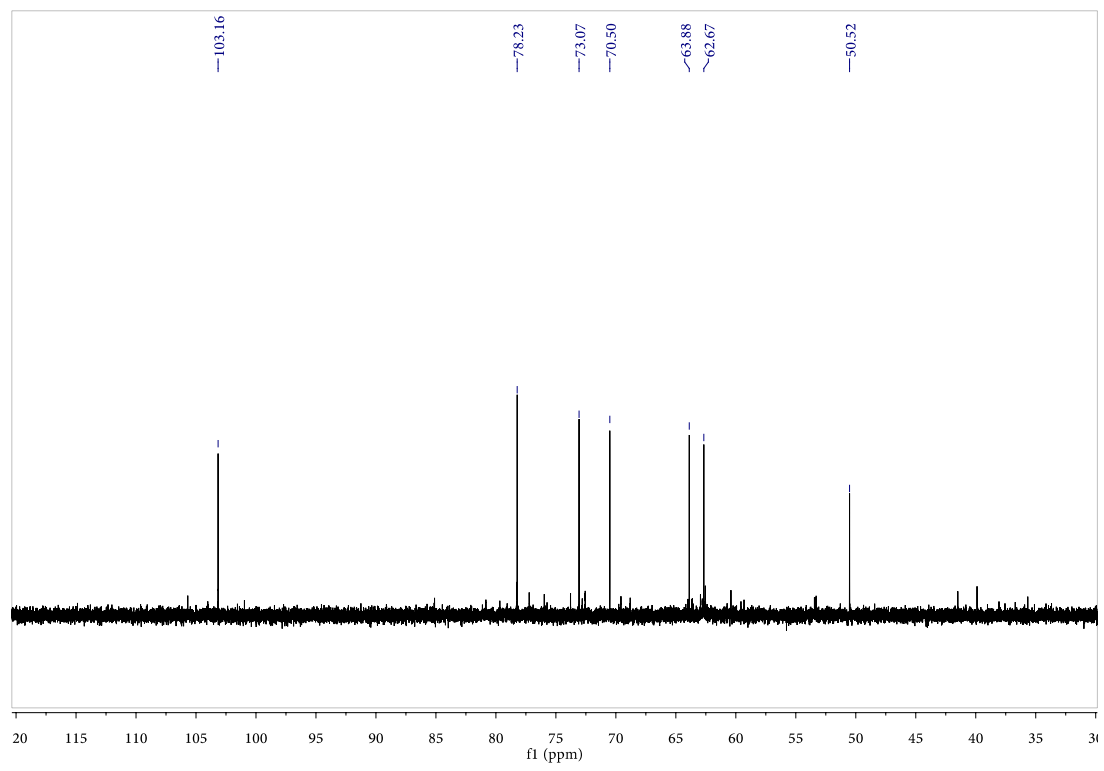


**b)**


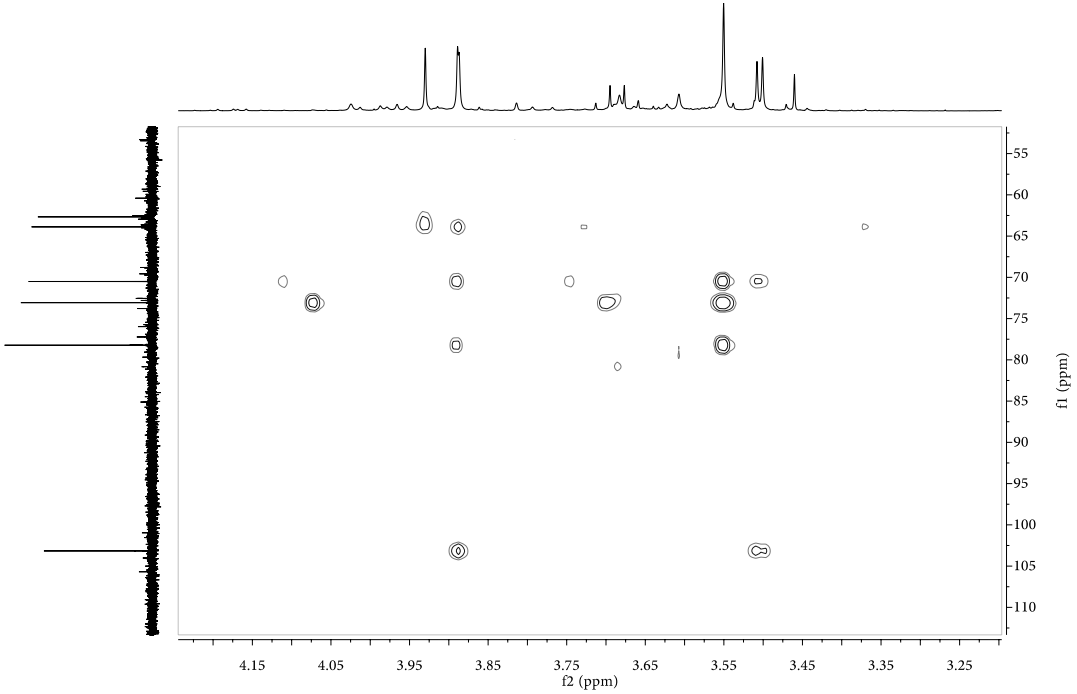


**c)**


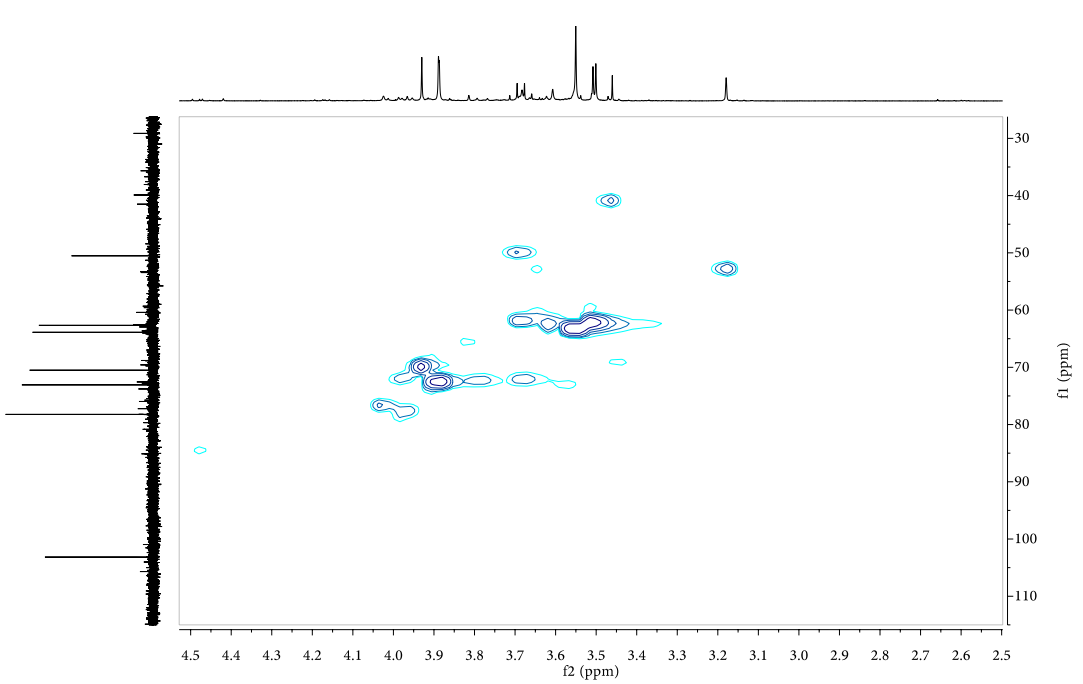


**d)**
